# Generation and Characterization of Adaptive Anoikis-Resistant Cells Using Cyclic Attachment-Detachment Culture of Cancer Cells

**DOI:** 10.21769/BioProtoc.5736

**Published:** 2026-07-05

**Authors:** Resha Rajkarnikar, Mehri Monavarian, Karthikeyan Mythreye

**Affiliations:** Division of Molecular Cellular Pathology, Department of Pathology, O’Neal Cancer Center, Heersink School of Medicine, The University of Alabama, Birmingham, AL, USA

**Keywords:** Anoikis, Matrix detachment, Cell death, Cell survival, Apoptosis, Spheroid, Adaptation

## Abstract

Anoikis resistance, or the ability of cancer cells to evade cell death triggered by immediate detachment from the extracellular matrix, is a critical established hallmark of metastatic cancer. While suspension culture models have been used to study anoikis, most focus on defined single time points or prolonged suspension that may not recapitulate the effects of repeated stress that tumor cells experience during metastatic dissemination. Here, we describe a detailed protocol for generating anoikis-resistant (AnR) cancer cells that have adapted to such stress through exposure to repeated cycles of suspension stress on poly-HEMA-coated plates, followed by recovery under standard attached conditions. The protocol includes methods for determining baseline anoikis sensitivity, generating AnR cells over 7–9 attachment-detachment cycles, assessing the stability and reversion of the anoikis-resistant phenotype, and characterizing AnR cells using Live/Dead staining of spheroids, flow cytometry–based apoptosis assays, and immunofluorescence for proliferation markers. This approach produces a non-genetic, reversible anoikis-resistant state that models the adaptive transcriptional reprogramming underlying metastatic progression, providing a reproducible and physiologically relevant in vitro system for studying anoikis resistance mechanisms and evaluating therapeutic strategies for prevention and reversal of such adaptations.

Key features

• Generates adapted anoikis-resistant cancer cells through cyclic attachment-detachment culture on poly-HEMA-coated plates over 7–9 passages.

• Produces a non-genetic and reversible resistant state that models adaptive transcriptional reprogramming during metastatic dissemination.

• Includes methods for assessing anoikis resistance stability, reversion kinetics, and cryopreservation of cells at defined passages.

• Provides three complementary characterization assays: Live/Dead spheroid imaging, Annexin V/PI flow cytometry, and Ki67 immunofluorescence.

## Graphical overview


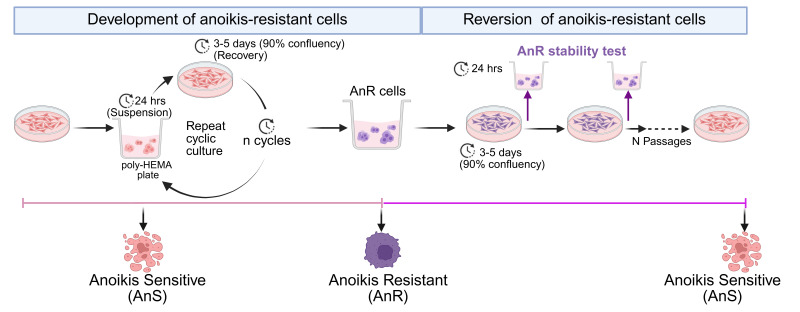


**Workflow and timeline for the generation of anoikis-resistant cells through alternating suspension and attached culture, followed by reversion to an anoikis-sensitive state by continuous passaging under attached conditions**

## Background

Anoikis, a form of programmed cell death triggered by loss of cell–extracellular matrix attachment, can serve as a critical barrier to metastasis. Since cell death is triggered rapidly, cancer cells that acquire anoikis resistance can survive during dissemination, enabling colonization of distant sites. Anoikis resistance is essential across diverse modes of metastatic spread. Circulating tumor cells (CTCs) in the bloodstream must withstand detachment to seed hematogenous metastases [1–3], while in ovarian and other peritoneal cancers, tumor cells must survive transit through the peritoneal cavity within malignant ascites to establish metastatic implants [4–7]. Despite its central role in metastasis across cancer types, methods for modeling the progressive acquisition of anoikis resistance in vitro remain limited.

Several approaches have been used to study anoikis resistance in vitro. These include culturing cells on ultra-low attachment surfaces for single time points, prolonged suspension culture to select for surviving populations, or the use of intrinsically anoikis-resistant cell lines [8–12]. While informative, single-time-point studies capture only the acute response to detachment stress and do not model the progressive adaptation that could occur during metastasis. Prolonged continuous suspension culture may select for preexisting resistant subpopulations rather than recapitulating the adaptive process. Furthermore, comparing intrinsically resistant lines to unrelated sensitive lines introduces confounding variables unrelated to anoikis adaptation.

The protocol described here addresses these limitations by generating adapted anoikis-resistant cells through repeated cycles of suspension stress followed by recovery under attached conditions, mimicking repeated cycles of detachment followed by attached regrowth/survival that tumor cells may experience during metastasis [13]. This approach produces an acquired, non-genetic anoikis-resistant state that is transient and reverts upon prolonged culture without suspension stress, consistent with transcriptional rather than mutational mechanisms of adaptation [13]. The model has been validated across multiple ovarian cancer cell lines with different baseline sensitivities to detachment stress and different mutational backgrounds [13]. The resulting anoikis-adapted cells display enhanced migration, chemoresistance, immune evasion, and metastatic potential in vivo [13], consistent with known associations of anoikis resistance and aggressive metastatic behavior [1,14–17]. The protocol is applicable to any adherent cancer cell lines where baseline anoikis sensitivity can be tested. The paired anoikis-sensitive (AnS) and anoikis-resistant (AnR) derivatives provide an isogenic system for studying the mechanisms underlying metastatic adaptation and for evaluating candidate therapeutics targeting this process.

## Materials and reagents

**Biological materials**

1. OV90 cells (ATCC, catalog number: CRL-11732)

2. CAOV3 cells (ATCC, catalog number: HTB-75)

**Reagents**

1. Medium 199, Earle’s salts (Thermo Fisher Scientific, Gibco, catalog number: 11150059)

2. MCDB 105 medium (Millipore Sigma, catalog number: 117-500)

3. DMEM (Corning, catalog number: MT10013CV)

4. FBS (Corning, catalog number: 35-011-CV)

5. Penicillin-Streptomycin (pen/strep) (10,000 U/mL) (Thermo Fisher Scientific, Gibco, catalog number: 15140122)

6. Poly (2-hydroxyethyl methacrylate) (poly-HEMA) (Millipore Sigma, catalog number: P3932)

7. Trypsin (2.5%) (Thermo Fisher Scientific, Gibco, catalog number: 15090046)

8. Trypsin EDTA (2.5%) (Millipore Sigma, catalog number: T4174-20ML)

9. Trypan Blue solution, 0.4% (Thermo Fisher Scientific, Gibco, catalog number: 15250061)

10. Dimethyl sulfoxide (DMSO) (Fisher Scientific, catalog number: BP231-100)

11. LIVE/DEAD^TM^ Viability/Cytotoxicity kit (Thermo Fisher Scientific, Invitrogen, catalog number: L3224)

12. Annexin V Apoptosis Detection kits (Thermo Fisher Scientific, eBioscience, catalog number: 88-8005-74)

13. Paraformaldehyde (PFA) (Fisher Scientific, J.T. Baker, catalog number: S898-07)

14. Ammonium chloride (NH_4_Cl) (Fisher Scientific, catalog number: AA11595A1)

15. Sodium hydroxide (NaOH) (Avantor, catalog number: MAL-7708-06)

16. Sodium chloride (NaCl) (Thermo Fisher Scientific, catalog number: S271-3)

17. Potassium chloride (KCl) (Thermo Fisher Scientific, catalog number: P217-500)

18. Sodium phosphate monobasic (NaH_2_PO_4_) (Millipore Sigma, catalog number: S0751-100G)

19. Potassium phosphate monobasic (KH_2_PO_4_) (Thermo Fisher Scientific, catalog number: P285-500)

20. Triton-X100 (Amresco, catalog number: 0694-1L)

21. Absolute ethanol (Fisher Scientific, catalog number: BP2818-4)

22. Bovine serum albumin (BSA) (Fisher Scientific, catalog number: BP9706100)

23. Ki-67 antibody (Cell Signaling, catalog number: 9449)

24. Goat anti-mouse IgG1 cross-adsorbed secondary antibody Alexa Fluor (Thermo Fisher Scientific, Invitrogen, catalog number: A-21125)

25. ProLong^TM^ Gold Antifade mountant (Thermo Fisher Scientific, Invitrogen, catalog number: P36930)

26. DAPI solution (Thermo Fisher Scientific, Invitrogen, catalog number: 62248)

**Solutions**

1. OV90 media (see Recipes)

2. CAOV3 media (see Recipes)

3. Poly-HEMA (see Recipes)

4. 4% PFA (see Recipes)

5. 10 mM NH_4_Cl solution (see Recipes)

6. 0.3% Triton-X 100 solution (see Recipes)

7. BSA (5% and 3%) (see Recipes)

8. 10× PBS (see Recipes)

9. FACS buffer (see Recipes)

**Recipes**

**1. OV90 media**

**Table:** 

Reagent	Final concentration	Quantity or volume
Medium 199, Earle’s salts	42%	42 mL
MCDB 105 medium	42%	42 mL
FBS	15%	15mL
Pen/strep	1%	1 mL
Total		100 mL

**2. CAOV3 media**

**Table:** 

Reagent	Final concentration	Quantity or volume
DMEM	89%	89 mL
FBS	10%	10 mL
Pen/strep	1%	1 mL
Total		100 mL

**3. Poly-HEMA**

**Table:** 

Reagent	Final concentration	Quantity or volume
poly-HEMA	2%	2 g
Absolute ethanol	95%	95 mL
Deionized (DI) water	5%	5 mL
Total		100 mL

Place the solution in the oven at 50 °C overnight. The next day, cool the solution to room temperature before using or store it at 4 °C for longer-term storage.

**4. 4% PFA**

**Table:** 

Reagent	Final concentration	Quantity or volume
PFA	4%	4 g
Distilled water		90 mL
10 N NaOH		~5 μL
10× PBS	1×	10 mL
Total		100 mL

Warm 80 mL of distilled water to 60 °C (do not boil) in a ventilation hood. Dissolve 4 g of PFA in the water by stirring. Slowly add 10 N NaOH (40 g of NaOH in 100 mL of DI H_2_O) using a dropper until the solution turns clear. Add 10 mL of 10× PBS. Adjust the final pH of the solution to 7.4. Make up the final volume of the solution to 100 mL. Filter the solution and store the excess in -20 °C.

**5. 10 mM NH_4_Cl solution**

**Table:** 

Reagent	Final concentration	Quantity or volume
NH_4_Cl	10 mM	21.4 mg
PBS (1×)		100 mL
Total		100 mL

**6. 0.3% Triton X-100 solution**

**Table:** 

Reagent	Final concentration	Quantity or volume
Triton X-100	0.3%	300 μL
PBS (1×)		100 mL
Total		100 mL

Sonicate the 0.3% Triton X-100 solution with two 15 s pulses. Prepare fresh solutions.

**7. BSA solution (5% and 3%)**

**Table:** 

Reagent	Final concentration	Quantity or Volume
BSA	5%	5 g
BSA	3%	3 g
PBS (1×)		100 mL
Total		100 mL

**8. 10× PBS**

**Table:** 

Reagent	Final concentration	Quantity or volume
NaCl	1.37 M	80 g
KCl	27 mM	2 g
Na_2_HPO_4_	100 mM	14.2 g
KH_2_PO_4_	18 mM	2.4 g
Total		1 L

Add all reagents to 900 mL of DI water. Adjust the pH between 7.2 and 7.4. Make up the total volume to 1,000 mL. For 1× PBS, add 100 mL of 10× PBS to 900 mL of DI water to make a total of 1 L.

**9. FACS buffer**

**Table:** 

Reagent	Final concentration	Quantity or volume
1× PBS		98 mL
FBS	2%	2 mL
Total		100 mL

**Laboratory supplies**

1. Tissue culture dishes (100 mm) (Fisher Scientific, catalog number: FB012924)

2. Tissue culture dishes (60 mm) (Fisher Scientific, catalog number: FB012921)

3. Tissue culture plates (6-well) (Fisher Scientific, catalog number: FB012927)

4. Millicell ultra-low attachment plate (96 well) (Millipore Sigma, catalog number: MC96ULA20)

5. 1,250 μL pipette tips (Fisher Scientific, catalog number: 12-111-010)

6. 200 μL pipette tips (Fisher Scientific, catalog number: 12-111-008)

7. 10 μL pipette tips (Fisher Scientific, catalog number: 12-111-006)

8. 10 mL serological pipettes (Fisher Scientific, catalog number: 14-955-234)

9. 25 mL serological pipettes (Fisher Scientific, catalog number: 14-955-235)

10. 15 mL conical tubes (Fisher Scientific, catalog number: 12-565-269)

11. 50 mL conical tubes (Fisher Scientific, catalog number: 12-565-271)

12. Hemocytometer (Reichert bright-line, catalog number: HS-1490)

13. 2 mL cryovial (Fisher Scientific, catalog number: 02-912-729)

14. Freezing container (Fisher Scientific, catalog number: 15-350-50)

15. Falcon round-bottom test tubes (FACS tubes) (Fisher Scientific, catalog number: 14-959-1A)

16. Cytoclip (Fisher Scientific, Epredia, catalog number: 59-910-052)

17. Cytofunnel (Fisher Scientific, Epredia, catalog number: 59-910-40)

18. Microscope slides (Fisher Scientific, catalog number: 22-037-246)

19. Coplin jars (Webber Scientific, catalog number: 2005-19)

20. PAP pen (Fisher Scientific, catalog number: NC9204359)

21. Micro cover glass (VWR, catalog number: 48366067)

## Equipment

1. Purifier class II biosafety cabinet (Labconco, catalog number: 3620904)

2. Centrifuge with rotors for 15 and 50 mL conical tubes (Eppendorf, catalog number: 5804)

3. Centrifuge with rotors for 1.5 mL microfuge tubes (Eppendorf, catalog number: 5424R)

4. Revco CO_2_ incubator (Thermo Electron, catalog number: 29584)

5. Convection oven (Thermo Scientific, catalog number: TS-PR305)

6. Vortex Genie 2 (Thermo Scientific, catalog number: 12-812)

7. Revco Elite Plus freezer (Thermo Scientific, catalog number: ULT1786-6-A43)

8. Liquid nitrogen tank (Thermo Scientific, catalog number: CY50985)

9. Refrigerator (Roper Scientific, model number: RT18DKXFW01)

10. Water bath (Polyscience, catalog number: NC1633292)

11. Inverted microscope (Nikon, catalog number: N-TS2)

12. Confocal microscope (Nikon, model: A1R HD)

13. Flow cytometer (BD LSRFortessa, UAB Flow Cytometry and Single Cell Core Facility)

14. Cytospin III (Shandon, catalog number: 8358-30-0001)

## Software and datasets

1. FlowJo software (BD Biosciences, version 10.8.1)

*Note: A license is required to use FlowJo. License options are available based on the number of users and frequency of use.*

2. ImageJ (NIH, version win64)

## Procedure

**A. Determination of anoikis sensitivity (AnS) of cells**

1. Plate 1–2 million cancer cells (ovarian cancer used here: OV90 or CAOV3) in a 100 mm dish in 10 mL of media (see Recipes 1 and 2).

2. Change the media every 3–4 days until confluency reaches 80%–90%.

**Critical:** Do not let confluency exceed 90%, as this can affect cell viability in suspension.

3. Prepare poly-HEMA-coated 6-well plates at least a day before plating cells in suspension by adding 1 mL of 2% poly-HEMA solution to each well and allowing it to dry at 50 °C overnight in the oven, followed by UV irradiation for 1 h before use (see Recipe 3).

*Note: Poly-HEMA-coated dried plates can be wrapped with parafilm and stored at 4 °C for future use.*

4. Aspirate the media from the plate and wash the cells with 5 mL of warm 1× PBS (see Recipe 8).

5. Add 2 mL of warm 1× trypsin EDTA and incubate cells in a 37 °C, 5% CO_2_ incubator until the cells detach from the plate.

6. Add 2 mL of full serum media to stop the trypsinization process and centrifuge the cells at 300× *g* for 5 min.

7. Resuspend the cells in 3–5 mL of media.

8. Make a 1:1 dilution of the cell solution and Trypan Blue and count the live cells by loading 10 μL of the solution into a hemocytometer.

*Note: Counting with a hemocytometer is preferred for AnS/AnR experiments for consistent results.*

9. Seed 250,000 live cells in each well of poly-HEMA-coated 6-well plates in 3 mL (Table 1) of complete growth media for 24–96 h.

*Note: For each time point, have a minimum of three wells as technical replicates.*

**Table 1.** Overview of experimental setup

Experiment	Plate format	Seeding density	Media volume
Development of AnR cells (suspension)	6-well plate	250,000	3 mL/well
Development of AnR cells (attached)	60 mm dish	500,000	4 mL
Characterization of AnS/AnR cells (single spheroids/well)	96-well ULA plate	1,000–2,500	200 μL
Characterization of AnS/AnR cells (aggregates)	6-well plate	250,000	3 mL/well

10. At each point, collect the cells in a 15 mL tube and centrifuge at 300× *g* for 5 min.

*Note: While collecting the cells, make sure the cells are not sticking to the cell culture plate. If a few cells are stuck, rinse the plate with an extra 1 mL of media and then collect the remaining cells in the tube.*

11. Aspirate the media and add 100 μL of warm 10× Trypsin EDTA and incubate at 37 °C with 5% CO_2_ for 5–10 min, depending on the tightness of the aggregates.

**Critica**l: Cells must be in a single-cell suspension for accurate viability assessment.

*Note: Both cell lines, OV90 and CAOV3, were incubated with 10× Trypsin EDTA for 10 min.*

12. After complete dissociation of cellular aggregates, add 400 μL of complete media to stop the trypsinization process.

13. Count the live cells as described in step A8.

14. Calculate the percentage of live cells for each time point and determine the time required to reach anoikis sensitivity for each cell line, where the relative live cell recovery compared to initial seeding is <100%.

*Notes:*

*1. The time point between 24 and 96 h for cells to exhibit anoikis (<100% live cells in suspension) for each cell line will be used for further experiments in section B.*

*2. Intrinsically anoikis-resistant cells will exhibit 100% or higher live cells in suspension and cannot be used further.*

**B. Generation of adapted anoikis-resistant (AnR) cells**

1. Plate 250,000 cells in each well of poly-HEMA-coated 6-well plates in 3 mL of complete media and maintain the cells in suspension for the duration required to reach the anoikis sensitive time point (step A14, see note 1).

**Critical:** Do not allow the confluency of cultured cells to exceed 90%, as this can affect cell viability in suspension.

*Note: The time required to keep cells in suspension is determined from step A14.*

2. Collect and count the cells as described in steps A9–13.

*Note: The cells collected from the first round of suspension are labeled P1_3D (Table 2).*

**Table 2.** Naming system for cells in attached and suspension passages

Culture	Passage number
First passage in suspension	P1_3D
Plating P1_3D cells in the attached condition for recovery	P1.1_2D
Plating P1.1_2D in suspension (second passage of suspension)	P2_3D
Plating anoikis-resistant cells in suspension after (n) cyclic passages	Pn_3D
Culturing Pn_3D cells in attached culture for reversal of anoikis resistance	Pn.1, Pn.2, Pn.3, ……Pn.N

3. Plate 500, 000 surviving cells in a 60 mm standard tissue culture dish with 4 mL of complete media (Table 1).

*Note: The cells cultured in attached condition after the first round of suspension are labeled P1.1_2D (Table 2).*

**Figure 1.**
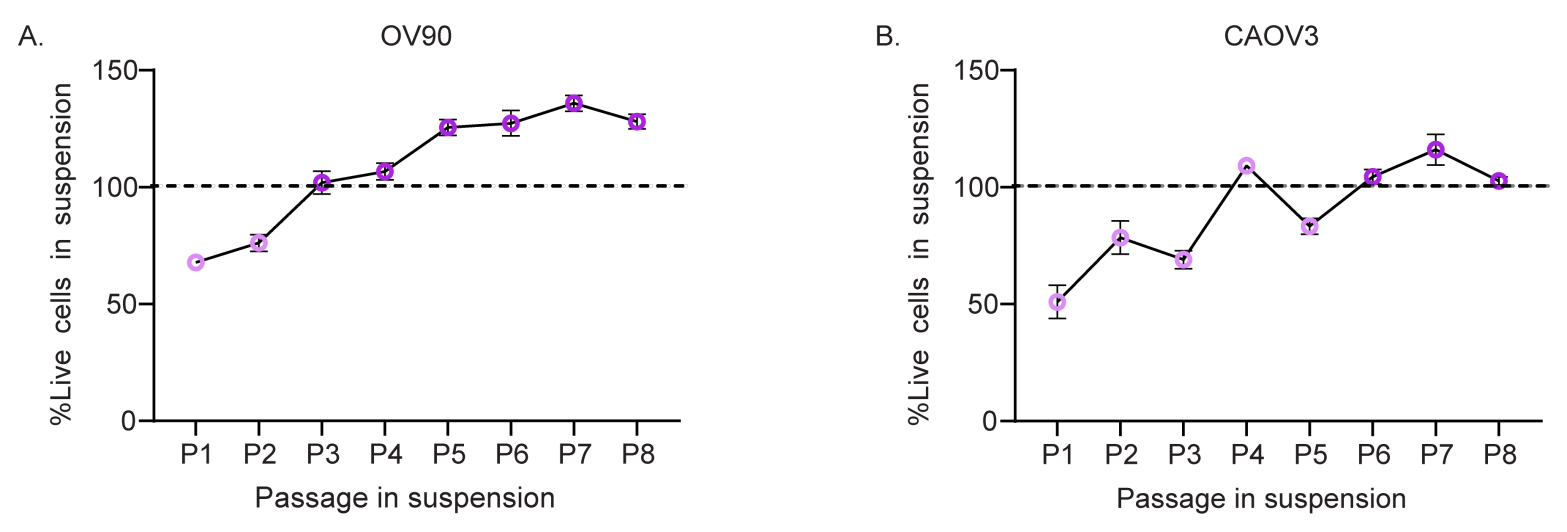
Generation of adapted anoikis-resistant cells. (A, B) Percentage of live cells of (A) OV90 and (B) CAOV3 relative to the initial seeding number, following 24 h in suspension, measured by Trypan Blue exclusion during repeated cycles of attachment and 24 h of suspension culture (N = 3–12). Data are mean ± SEM.

4. Change the media every 2–3 days and allow the cells to grow until 90% confluency.

5. Repeat steps B1–4 of culturing cells in suspension, followed by recovery for 6–8 additional cycles or until the population reaches and maintains 100% or higher live cell recovery compared to initial seeding in suspension for at least three cycles.

6. Calculate the percentage of live cells for each passage in suspension to determine the cyclic passages required to reach anoikis resistance (≥100% relative cell recovery compared to initial seeding) (Figure 1).

*Notes:*

*1. The suspension passages where live cell recovery is <100% compared to the initial seeding number are considered anoikis sensitive (AnS); a relative live cell recovery ≥100% compared to initial seeding is considered anoikis resistant (AnR).*

*2. While stable AnR is defined here by maintenance of ≥100% relative live cell recovery in suspension compared to initial seeding for at least three additional cycles, this may vary by cell line.*

**C. Stability of adapted anoikis resistance in cells**

1. Collect the adapted anoikis-resistant cells cultured in suspension as described in steps A10–12.

2. Count the cells using Trypan Blue exclusion.

3. Plate 500,000 live cells in a 60 mm dish in 4 mL of complete growth medium until it reaches 90% confluency.

*Note: The first passage of AnR cells (e.g., P7) in 2D can be labeled as AnR P7.1 2D.*

4. On the day of splitting, aspirate the media from the plate and wash the cells with 2 mL of warm 1× PBS.

5. Add 1 mL of warm 1× Trypsin with EDTA and incubate the cells at 37 °C with 5% CO_2_ for 2–10 min until the cells dissociate from the plates.

6. Add 1 mL of full serum media to stop the trypsinization process and centrifuge the cells at 300× *g* for 5 min.

7. Resuspend the cells in 3–4 mL of media and count the live cells.

8. Count the number of live cells as mentioned in step A8 and keep note of the total number of live cells.

9. Seed 250,000 cells from the cell solution in each well of poly-HEMA-coated 6-well plates in 3 mL of complete growth media and maintain the cells in suspension for the duration required to reach the anoikis sensitive time point as calculated from step A14.

10. Plate 500,000 cells from the remaining cell solution in a 60 mm dish in 4 mL of complete growth medium until it reaches 90% confluency.

*Note: The following AnR cell passages in 2D can be labeled as P7.2 2D, P7.3 2D, etc.*

**Figure 2.**
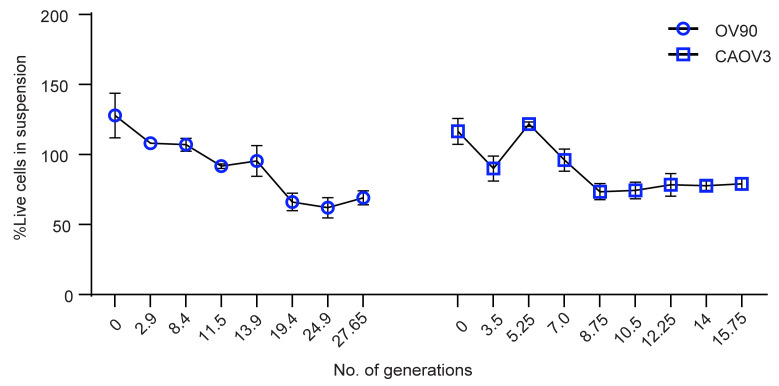
Stability of adapted anoikis-resistant cells. Percent live OV90 and CAOV3 AnR cells relative to the initial seeding number upon challenge with suspension stress for 24 h after attached cell culture expansion, plotted against the number of generations in attached culture at the time of suspension stress challenge. Data are mean ± SD.

11. After the cells (seeded at step C9) reach their suspension time point, collect and count the live cells as described in step A10–A12.

12. After the cells cultured in 2D (step C10) conditions reach 90% confluency, passage the cells by repeating steps C4–10 for a total of 9–11 passages; in each passage, challenge the cells to suspension stress as described in step C9.

13. Calculate the number of generations using the formula: generation = log_2_(x), where x equals the number of live cells divided by the number of cells plated in the attached condition.

14. Plot a graph with the number of generations (step C13) on the x-axis and the percentage of live cells in suspension (step C11) on the y-axis to identify the number of generations required to revert anoikis resistance in cells (Figure 2).

**D. Cryopreservation/thawing of adapted anoikis-resistant cells**

1. For cryopreservation:

a. Make a cell solution of 1–2 million/mL in complete growth media.

b. Add 10% DMSO to the cell solution and carefully pipette up and down to mix the cell solution and transfer the cell solution to a prelabeled cryovial.

*Note: Label the passage of the cells on the cryovials, indicating if collected from suspension or attached condition, as described in Table 2.*

c. Transfer vials to the freezing container and store at -80 °C overnight, followed by storage in the liquid nitrogen tank.

2. Thawing AnR cells:

a. For each cryovial, prepare a 15 mL conical tube with 9 mL of complete growth media at 37 °C.

b. Remove the cryovial from the liquid nitrogen tank and thaw the cells in 37 °C until only a pea-sized amount of ice is left (approximately 1 min).

c. Add 0.5 mL of media prepared in the conical tube to the cryovial, dissolve the remaining ice, and transfer the entire cell solution to the conical tube.

d. Centrifuge the cells at 300× *g* for 5 min.

e. Remove the supernatant and resuspend the cells in 1–3 mL of complete media at 37 °C.

f. Count the number of live cells and plate them in a tissue culture plate.

*Note: If thawing the cells that were frozen directly from 3D culture, e.g., P1 3D, after thawing, label them as P1.1 2D.*

g. Once the cells reach 80%–90% confluency, plate the cells in suspension as in steps B1 and B2 and calculate the percentage of live cells in suspension to test anoikis sensitivity/resistance before proceeding.

**E. Analysis of the LIVE/DEAD ratio in adapted anoikis-resistant cells in spheroids by Calcein-AM and red fluorescent ethidium homodimer-1 staining**

1. Culture AnS and AnR cells in 2D culture until they reach 90% confluency.

2. Plate 1,000–2,500 cells in 200 μL (Table 1) complete media in each well of an ultra-low attachment U-bottom 96-well plate.

3. Centrifuge the plate at 300× *g* for 2 min.

*Notes:*

*1. Balance the plate with another plate containing the same volumes while centrifuging.*

*2. After the centrifugation is complete, make sure all cells are at the center of the wells and look equal in numbers.*

4. Incubate the plates at 37 °C with 5% CO_2_ for 24 h.

5. After 24 h, remove 100 μL of media from each well and add 100 μL of warm 1× PBS at 37 °C.

**Critical:** When removing the media from the well, pipette the solution without disturbing the spheroids. Angle the tip on the wall of the well and gently remove the solution. Do not pipette up and down after adding PBS.

*Note: Retain 100 μL of solution in the wells at all times.*

6. Centrifuge the plate at 300× *g* for 2 min.

7. Remove 100 μL of solution from each well.

**Critical:** Carefully pipette the solution from the well without disturbing the spheroids.

8. Remove the LIVE/DEAD assay reagent from the freezer and allow it to warm to room temperature.

9. Prepare the green fluorescent Calcein-AM and red fluorescent ethidium homodimer-1 from the LIVE/DEAD^TM^ Viability/Cytotoxicity kit in 100 μL of PBS at 37 °C. Add the staining solution to each well to obtain a final concentration of 4 μM Calcein-AM and 8 μM ethidium homodimer-1 in a total volume of 200 μL.

*Note: If the cells are already fluorescently labeled, pick Calcein-AM and ethidium homodimer-1 that do not overlap with the excitation and emission spectra of the fluorescence in the cells.*

10. Add 100 μL of warm PBS containing the LIVE/DEAD assay reagent for 30–45 min.

*Note: The optimal concentration of the LIVE/DEAD reagents and the duration of incubation will depend on the cell line and the degree of spheroid compaction. Optimize the concentration and duration to achieve the lowest dye concentration and the shortest duration that yields the maximum signal.*

**Figure 3.**
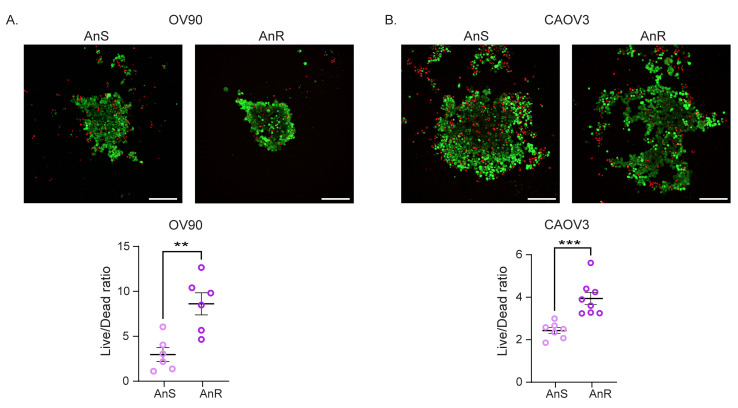
Analysis of the LIVE/DEAD ratio of adapted anoikis-resistant cells in spheroids. (A, B) Representative images of OV90 (A, 1,000 cells) and CAOV3 (B, 2,500 cells) anoikis-sensitive (AnS) and anoikis-resistant (AnR) single spheroids cultured in ultra-low attachment plates in suspension for 24 h, stained with Calcein AM (green, live cells) and ethidium homodimer (red, dead cells). Scale bars, 200 μm. Respective data (below) is shown as the Live/Dead ratio. Data shown as mean ± SEM, unpaired Student’s t-test, ** p < 0.01, *** p < 0.001 (n = 6).

11. Centrifuge the plate at 300× *g* for 2 min.

12. Carefully remove 100 μL of solution from each well.

13. Wash the cells three times with 100 μL of warm 1× PBS at 37 °C.

14. Image the spheroids using confocal microscopy (Figure 3).

15. Analyze the images for the live/dead ratio by calculating corrected total fluorescence (CTCF) for the Calcein-AM and ethidium homodimer-1 fluorescence channels using ImageJ software.

**F. Analysis of apoptosis of adapted anoikis-resistant cells by flow cytometry**

1. Plate 250,000 Ans and AnR cells in each well of poly-HEMA-coated 6-well plates in 3 mL of complete growth media for 24 h.

2. After 24 h, collect the cells and trypsinize as described in steps A10–12.

**Critical:** For the apoptosis assay, use trypsin without EDTA, since EDTA can interfere with annexin V staining.

3. Count the cells using Trypan Blue exclusion.

**Critical:** Make sure the cells are trypsinized completely and are single cells.

4. Transfer the cells into a microfuge tube and make the total volume up to 1 mL with the complete media.

5. Centrifuge the cells at 360× *g* for 5 min.

6. Carefully aspirate the media without disturbing the pellet.

7. Add 1 mL of 1× cold PBS and mix by pipetting.

8. Centrifuge the microfuge tubes at 360× *g* for 5 min.

9. Repeat the wash with 1× cold PBS.

10. Prepare 1× binding buffer in 1× cold PBS by diluting the 10× binding buffer provided with the Annexin V Apoptosis Detection kit.

11. Remove the PBS from the microfuge tubes and add 1 mL of cold 1× binding buffer and mix gently.

12. Centrifuge the microfuge tubes at 360× *g* for 5 min.

13. Aspirate the solution and resuspend the cells in cold 1× binding buffer at a concentration of 1 million cells/mL (or 100,000 cells/100 μL).

14. Add 5 μL of fluorochrome-conjugated Annexin V to 100 μL of cell solution, mix gently, and incubate in the dark for 15 min.

*Note: All steps from here should be performed in the dark.*

15. Centrifuge the microfuge tubes at 360× *g* for 5 min.

16. Gently aspirate the solution with the pipette, add 1 mL of 1× cold binding buffer to the microfuge tubes, and mix gently.

**Critical:** Do not use suction to aspirate, as it can result in the loss of the cells.

17. Centrifuge the microfuge tubes at 360× *g* for 5 min.

18. Aspirate the solution and resuspend the cells in 100 μL of cold 1× binding buffer.

19. Add 5 μL of propidium iodide staining solution included in the Annexin V Apoptosis Detection kit to each microfuge tube.

**Critical:** Do not wash the cells after staining with propidium iodide, which could result in loss of signal.

20. Label the FACS tube for each sample and add 200 μL of FACS buffer (see Recipe 9).

21. Transfer the stained cells to the FACS tube and analyze the cells by flow cytometry.

*Note: Cells can be analyzed within 4 h of staining by storing them at 4 °C in the dark.*

**Figure 4.**
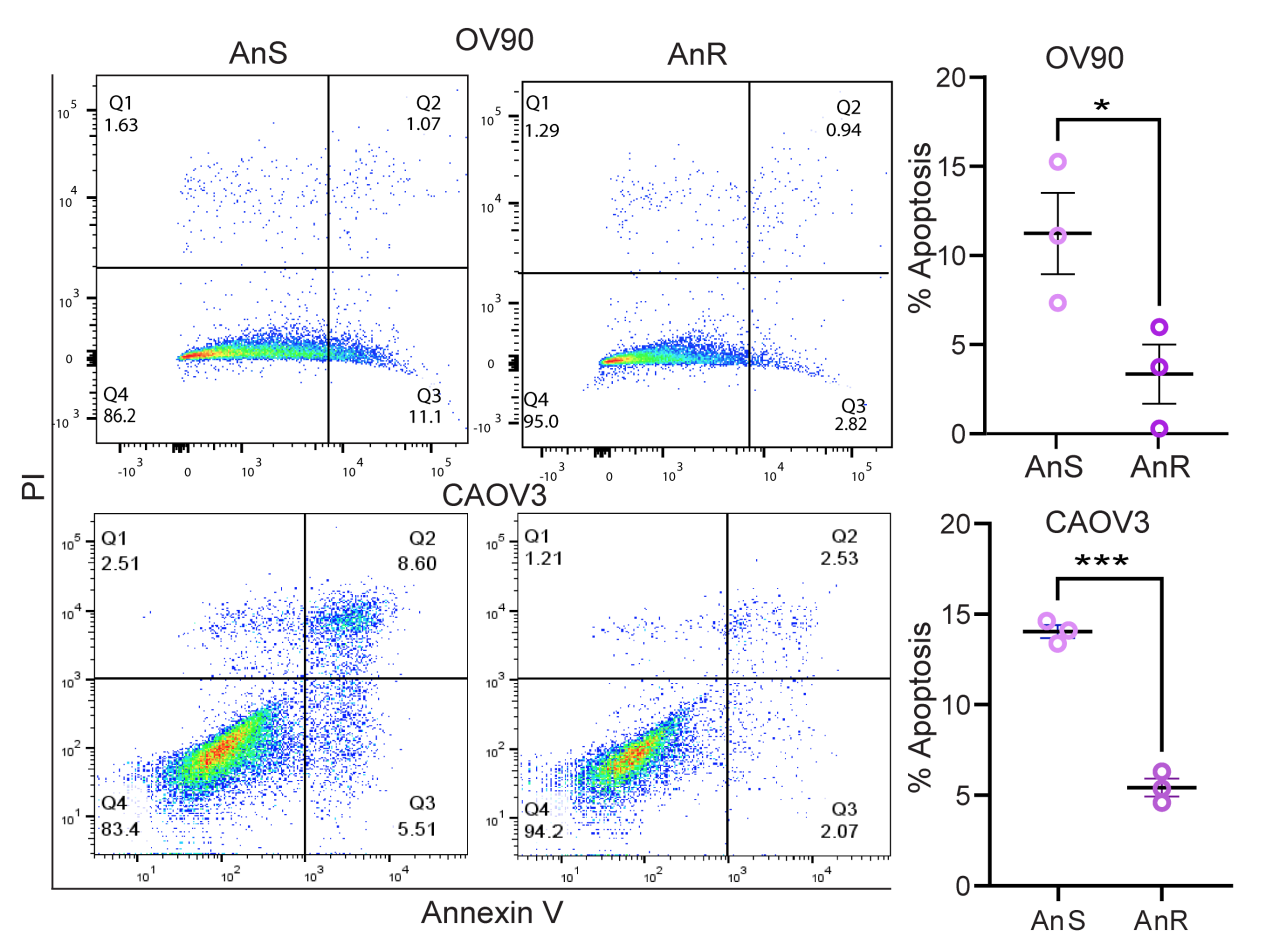
Analysis of apoptosis of adapted anoikis-resistant cells by flow cytometry. Representative flow cytometry data (left) of OV90 and CAOV3 anoikis-sensitive (AnS) and anoikis-resistant (AnR) cells cultured in suspension for 24 h and stained with Annexin V and propidium iodide (PI). Quantification of percent apoptosis (right) analyzed as mean ± SEM, unpaired Student’s t-test, *p < 0.05, ***p < 0.001 (n = 3).

22. Analyze the data using FlowJo software (Figure 4).

**G. Analysis of proliferation of adapted anoikis-resistant cells by Ki67 staining using immunofluorescence**

1. Plate 250,000 AnS and AnR cells in each well of poly-HEMA-coated 6-well plates in 3 mL of complete growth media for 24 h.

2. After 24 h, collect the cells in 15 mL centrifuge tubes and centrifuge at 300× *g* for 5 min.

3. Aspirate the media and add 400 μL of cold 1× PBS and transfer the cell solution to a microcentrifuge tube.

4. Centrifuge the cells at 360× *g* for 2 min, then aspirate the solution without disturbing the cell pellet.

5. Add 400 μL of 4% PFA (see Recipe 4) for 15 min at room temperature.

6. Wash the cells with cold 1× PBS twice.

7. Add 400 μL of 10 mM NH_4_Cl (see Recipe 5) for 5 min at room temperature.

8. Centrifuge the cells at 360× *g* for 2 min, then aspirate the solution without disturbing the cell pellet.

9. Wash the cells with 400 μL of cold 1× PBS for 5 min.

10. Centrifuge the cells at 360× *g* for 2 min, then aspirate the solution without disturbing the cell pellet.

11. Permeabilize the cells with freshly made 400 μL of 0.3% Triton X-100 (see Recipe 6) for 10 min at room temperature.

12. Centrifuge the cells at 360× *g* for 2 min, then aspirate the solution without disturbing the cell pellet.

13. Wash the cells with 400 μL of cold 1× PBS twice for 5 min.

14. Block the cells with 400 μL of 5% BSA solution made in 1× cold PBS (see Recipe 7) and incubate for 1 h at room temperature.

15. Centrifuge the cells at 360× *g* for 2 min, then aspirate the solution without disturbing the cell pellet.

16. Wash the cells with 400 μL of cold 1× PBS for 5 min.

17. Add 100 μL of Ki-67 antibody at a dilution of 1:450 made in 3% BSA (see Recipe 7) and incubate overnight at 4 °C on a shaker.

18. Centrifuge the cells at 360× *g* for 2 min, then aspirate the solution without disturbing the cell pellet.

19. Wash the cells with 400 μL of cold 1× PBS for 5 min three times.

20. Add 100 μL of goat anti-mouse IgG1 cross-adsorbed secondary antibody Alexa Fluor at a 1:500 dilution made in 3% BSA (see Recipe 7) and incubate at room temperature for 1 h in the dark.

21. Centrifuge the cells at 360× *g* for 2 min, then aspirate the solution without disturbing the cell pellet.

22. Wash the cells with 400 μL of cold 1× PBS for 5 min three times.

23. Stain the nuclei with 100 μL of DAPI solution at a dilution of 1:2,000 of the 1 mg/mL stock solution made in 1× cold PBS and incubate for 10 min at room temperature in the dark.

24. Centrifuge the cells at 360× *g* for 2 min and wash the cells with 400 μL of cold 1× PBS for 5 min.

25. Resuspend the cell pellet in 200 μL of cold 1× PBS.

26. Label the charged microscope slides and place them in the cytoclip, followed by a cytofunnel.

*Note: Label on the frosted side of the slides.*

**Critical:** Make sure the hole in the filter card and the cytofunnel are aligned before loading the cytoclip onto the cytospin.

**Figure 5.**
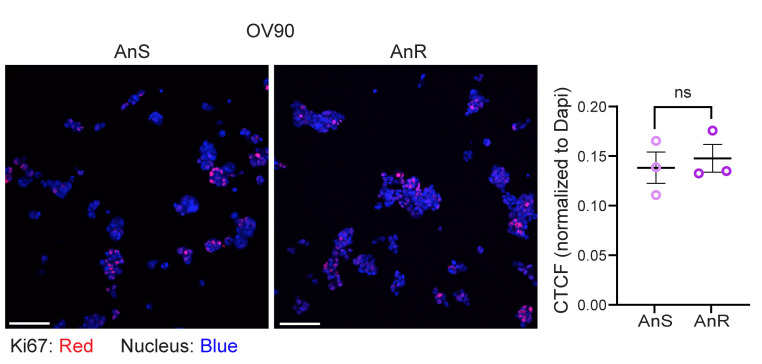
Analysis of proliferation of adapted anoikis-resistant cells by Ki67 staining using immunofluorescence. Representative images (left) of OV90 anoikis-sensitive (AnS) and anoikis-resistant (AnR) cells cultured in suspension for 24 h, cytopsun, and stained with Ki67 and DAPI. Scale bar, 100 μm. Quantification (right) of corrected total fluorescence (CTCF) of ki67-normalized to DAPI. Data are mean ± SEM; unpaired Student’s t-test; ns, non-significant (n = 3).

27. Load 100 μL of cell solution onto each slide through the opening of the cytofunnel.

28. Cytospin the slides at 100× *g* for 5 min.

29. Add Prolong Gold Antifade mounting media on top of the cells and gently lower a coverslip at an angle to avoid bubbles.

30. Let the mounting media dry and image the cells using a confocal microscope (Figure 5).

31. Analyze the images using ImageJ software.

## Validation of protocol

This protocol has been used and validated in the following research article:

• Monavarian and Rajkarnikar et al. [13]. Anoikis resistance and metastasis of ovarian cancer can be overcome by CDK8/19 mediator kinase inhibition. *JCI Insight*. (Figure 1C, E, F, H and Figure 2D). https://doi.org/10.1172/jci.insight.192113

## General notes and troubleshooting

**General notes**

1. When counting cells for either plating the experiments or for counting live cells in suspension, be consistent with the counting method (manual vs. automatic counter).

2. When coating the poly-HEMA plates, make sure the coating is even and dried completely; otherwise, it could lead to attachment of cells in uncoated areas.

**Troubleshooting**

**Problem 1:** Cells cultured in suspension for >24 h form tight aggregates and are difficult to dissociate into single cells for counting.

Possible cause: Strong cell–cell junction.

Solution: Every 2–3 min, remove the cells from the incubator and mechanically disrupt the aggregates using a P1000 pipette tip, applying 5–10 firm strokes.

**Problem 2:** Cells do not recover well after the suspension.

Possible cause: Insufficient number of cells.

Solution: Plate more than 500,000 cells in a 60 mm dish if needed. However, optimize the number of cells so that the cells in recovery can undergo at least two doubling-time points.

**Problem 3:** Cell contamination during long cyclic culture.

Possible cause: Non-aseptic cell culture practices, poly-HEMA is not sterile.

Solution: Use clean gloves and sterile reagents and work in hoods and incubators when handling cells. If using poly-HEMA plates stored at 4 °C, make sure to expose them to UV light for at least 1 h before use.
